# Predicting completion and early dropout in routine-care internet-delivered cognitive behaviour therapy: A temporal validation study

**DOI:** 10.1016/j.invent.2026.101011

**Published:** 2026-10-12

**Authors:** Ram P. Sapkota, Blake F. Dear, Nickolai Titov, Heather D. Hadjistavropoulos

**Affiliations:** a Department of Psychology, University of Regina, 3737 Wascana Parkway, Regina, SK, S4S 0A2, Canada; b eCentreClinic, School of Psychological Sciences, Macquarie University, Sydney, NSW, 2109, Australia; c MindSpot Clinic, School of Psychological Sciences, Macquarie University, Sydney, NSW, 2109, Australia

**Keywords:** ICBT, Engagement, Completion, Early dropout, Temporal validation

## Abstract

**Background:**

In routine-care Internet-delivered cognitive behaviour therapy (ICBT), engagement may be better understood as a staged process (e.g., early discontinuation vs full completion), yet many studies use a single dropout definition and seldom test whether prediction models remain useful as services evolve over time.

**Objective:**

To identify baseline predictors of completion and early dropout in routine-care ICBT and evaluate temporal transportability from earlier to later service years.

**Methods:**

We analyzed de-identified routine-care data (*N* = 9057) from a transdiagnostic ICBT program delivered from 2013 to 2024. Outcomes were completion (all five lessons vs fewer) and early dropout (0–1 lesson vs two or more). Multivariable logistic regression models used baseline demographic, clinical, and contextual intake variables. Models were developed in TRAIN (2013–2021) and applied without refitting to TEST (2022–2024). TEST performance was summarized using Brier score, area under the curve (AUC), and calibration.

**Results:**

Demographic factors, including age, education, ethnicity, and relationship status, were more consistently associated with engagement than baseline symptom severity. Completion predictions showed modest discrimination and modest overprediction in TEST, whereas early-dropout predictions showed weaker discrimination, slight underprediction, and limited separation of higher- and lower-risk clients.

**Conclusions:**

Models based on screening variables showed higher discrimination for completion than early dropout. Engagement prediction in routine-care ICBT should be temporally evaluated and monitored for calibration drift. Future research should examine whether early indicators during treatment, such as initial logins, lesson access, and early therapist contact, improve prediction of early dropout.

## Highlights

• Completion and early dropout showed different predictor patterns.
• Demographic factors predicted engagement more than symptom severity.
• Performance and calibration shifted in temporal validation.
• Identifying early dropout may require in-treatment signals beyond screening.

## 1 Introduction

Internet-delivered cognitive behaviour therapy (ICBT) is an evidence-based intervention modality for common mental disorders such as depression and anxiety. Large syntheses continue to support its efficacy across adult mental disorders (Cuijpers et al., 2025; Hedman-Lagerlöf et al., 2023), including in routine-care contexts (Etzelmueller et al., 2020). However, as in face-to-face therapy (Fernandez et al., 2015; Kullgard et al., 2022; Swift and Greenberg, 2012) engagement remains a primary implementation constraint in digital care, with many users not completing the full program, and a subset disengaging early (Bisby et al., 2022; Cross et al., 2022). In routine care, non-completion does not necessarily indicate treatment failure, as some clients stop because they feel sufficiently improved, the program is a poor fit for their needs or preferences, or because life events and competing demands make it difficult to continue. From a service perspective, the aim is not necessarily completion for every client. At the same time, engagement is not only a patient-level phenomenon; it can also reflect modifiable features of intervention and service design, including how digital programs are structured, supported, and integrated into routine workflows (McCall et al., 2021; Smith et al., 2025).

Digital interventions commonly show substantial loss of patients at multiple points, ranging from non-initiation to early discontinuation and partial completion. This pattern is often discussed in terms of Eysenbach's (2005) “law of attrition,” which highlights that drop-off is expected in digital programs and can occur at different stages; the concept describes the general pattern of attrition rather than implying any specific attrition rate for a given service. Yet attrition is not a single construct, and collapsing engagement into one “dropout” indicator can obscure distinct phenomena with different determinants and service implications. A recent meta-analysis comparing internet-based and face-to-face cognitive behaviour therapy distinguishes treatment non-initiation, dropout after starting, and broader attrition, underscoring the need to operationalize engagement as a staged process rather than a single endpoint (Linardon et al., 2025). Conceptual work on adherence in eHealth similarly emphasizes that “use” becomes “adherence” only relative to intended use, which varies by program design and by the clinical or service objective (Sieverink et al., 2017). Consistent with this, engagement appears heterogeneous and may cluster into distinct usage trajectories rather than a single completer vs dropout pathway (Chien et al., 2020), and qualitative work suggests discontinuation can reflect practical barriers, shifting motivation, or early experiences with the program and support rather than symptom severity alone (Lawler et al., 2021; Peynenburg et al., 2022). This literature suggests that disengagement is heterogeneous: when clients stop may matter as much as whether they stop, and the reasons for stopping may not be well captured by symptom severity alone.

Predictor research in ICBT has likewise been limited by inconsistent outcome definitions and analytic approaches that may not capture the full complexity of engagement. In routine care and pragmatic delivery settings, engagement may be shaped by patient characteristics (e.g., age and sociodemographic factors), clinical presentation, and service features (e.g., onboarding, support intensity, platform design) and external circumstances outside the service that can affect a person's ability to continue. Emerging work suggests that predictors may differ depending on how engagement is operationalized. For example, a secondary analysis of a web-based CBT program for primary-care patients with depression or anxiety symptoms examined predictors of program use and dropout, highlighting the need to distinguish different engagement endpoints rather than treating attrition as a single outcome (Rotondi et al., 2024). Related work in blended and digitally supported CBT similarly demonstrates that non-initiation and dropout can be predicted by different combinations of sociodemographic and clinical features (Wu et al., 2022). More recently, Zantvoort et al. (2024) used demographic, symptom, and online activity data collected during the first four weeks of routine-care ICBT to predict subsequent dropout, illustrating the potential value of in-treatment information in addition to baseline characteristics.

Recent personalization frameworks for ICBT also distinguish treatment-entry stratification from responsiveness during treatment, emphasizing that engagement and clinical outcomes are related but distinct targets for personalization (Aafjes-van Doorn and Christensen, 2026). In routine care, adherence modelling in guided ICBT suggests that engagement prediction can inform implementation planning, for example by helping services plan targeted outreach and support intensity (Österman et al., 2024). At the intervention level, some evidence suggests that program features intended to support engagement (e.g., persuasive design elements) may be associated with differences in outcomes in unguided ICBT, indicating that engagement is not solely a patient attribute but can also reflect modifiable aspects of intervention and service delivery (McCall et al., 2021; Peynenburg et al., 2022). Across studies, however, predictor patterns are not fully consistent and appear to vary depending on how engagement is defined and, on the population, and service context.

Within therapist-assisted ICBT, Edmonds et al. (2018) showed that baseline patient characteristics help explain who benefits and who drops out, but engagement was largely treated as a single dropout construct within a narrower service window. Building on this foundation, the present study conceptualizes engagement as staged (early dropout vs full completion) and evaluates temporal transportability by testing whether models developed in earlier service years retain performance when applied, without refitting, to later service years. This emphasis is important because prediction models can degrade when patient mix, workflows, or measurement practices shift over time. Evaluating performance under realistic, earlier-years → later-years deployment, conditions is now recognized as good practice in applied prediction (Bennemann et al., 2022; Collins et al., 2024).

A further gap limits the usefulness of the engagement literature for service planning. Many predictor studies are developed and interpreted within a single time period, without explicitly examining whether findings from earlier years still apply in later years of service delivery. This matters in digital programs that evolve over time, including changes in assessment scheduling, recruitment procedures, therapist workflows, and client mix. When these conditions change, conclusions based on earlier years may not hold in later years, so later-year evaluation is essential for practical service planning (Bennemann et al., 2022; Collins et al., 2024). The present study addresses these gaps using a large routine-care sample from a provincial ICBT service spanning more than a decade of delivery.

Engagement was operationalized using two binary outcomes derived from lesson-completion records: treatment completion (completion of all five core lessons) and early dropout (discontinuation after 0–1 lesson versus engagement through two or more lessons), consistent with staged conceptualizations of engagement and attrition (Eysenbach, 2005; Linardon et al., 2025; Sieverink et al., 2017). To examine whether findings based on earlier service years remained applicable to later service years, we also evaluated the temporal transportability of the engagement prediction models (Collins et al., 2024).

We focused on two clinically meaningful engagement endpoints: treatment completion and early dropout. By distinguishing these engagement stages and examining whether patterns observed in earlier years also apply in later years, this study aimed to clarify which screening factors are most consistently linked to completion and early dropout in routine-care ICBT and to inform service planning and practical engagement supports. This focus is directly relevant to decisions about the timing and intensity of early contact and whether additional support is offered universally or targeted to clients at higher risk of early disengagement. Targeted contact can improve adherence in some guided digital interventions (Pihlaja et al., 2020). However, disengagement may also reflect factors outside the service and will not always be preventable, so understanding reasons for stopping and identifying actionable early indicators remain important directions for future work. Because engagement patterns and service delivery features can vary across settings, the findings may not generalize directly to other service settings. However, they provide a practical example of how engagement patterns and their correlates can shift across implementation eras in a long-running routine-care service.

## 2 Methods

### 2.1 Design and data source

The Online Therapy Unit (OTU) provides provincially funded, therapist-assisted, transdiagnostic ICBT as routine clinical care in Saskatchewan (Hadjistavropoulos and Peynenburg, 2025). OTU maintains an ongoing program evaluation dataset compiled from standard screening and baseline questionnaires, as well as treatment-platform records. As part of routine service delivery, clients are invited to provide informed consent for research use of their data. All procedures have been approved by the University of Regina Research Ethics Board under a series of protocols that support continuous routine-care data collection as program operations and research aims have evolved over time. This study is a secondary analysis of fully de-identified routine-care data pooled across program delivery years; because the analyses used de-identified archival data collected under existing approvals, additional ethics approval was not sought. Data from this program have also supported prior publications (e.g., Hadjistavropoulos et al., 2022; Roemmel et al., 2025) but the current study addresses a different research objective focused on engagement prediction.

Data were drawn from routine delivery of the transdiagnostic Wellbeing Course delivered by OTU between November 2013 and December 2024. The analytic file included participants with observed engagement outcomes and complete data on the predictors used in the primary model specification. Consistent with staged conceptualizations of engagement and attrition (Eysenbach, 2005; Linardon et al., 2025; Sieverink et al., 2017), engagement outcomes were defined as (1) treatment completion, operationalized as completing five lessons versus fewer than five lessons, and (2) early dropout, operationalized as completing 0–1 lesson versus completing two or more lessons. A major program-era change occurred in assessment scheduling: from 2013 to 2019, symptom measures were administered in a lesson-anchored manner, whereas from 2020 onward they were administered weekly irrespective of lesson completion. This assessment-administration shift was used to contextualize engagement patterns over time.

To evaluate whether engagement prediction models developed in earlier service years remained accurate when applied to later service years, we used a prespecified time-based validation design. Models were estimated using screening data from 2013 to 2021 (TRAIN) and then applied unchanged to screening data from 2022 to 2024 (TEST). The three-year TEST window was selected in advance because early dropout was uncommon; pooling three years increased the number of early-dropout events and reduced sensitivity to chance variation or any single year that may have been atypical due to broader system or service changes, including the COVID-19 period. We did not assume that 2022, 2023, and 2024 were equivalent years; year-specific engagement rates are shown in Supplementary Fig. S1 to contextualize the evaluation window. This split also aligns with prior OTU prediction work that analyzed data through 2021 (e.g., Hadjistavropoulos et al., 2022; Roemmel et al., 2025), leaving 2022 to 2024 as a distinct post-2021 evaluation period. In the 2022–2024 TEST period, we evaluated discrimination, overall accuracy, and calibration (agreement between predicted and observed outcome rates), and pooled scoring summaries are also reported to summarize overall performance, although temporal TEST results provide the main evidence of transportability (Collins et al., 2024). Further details of the temporal validation procedures and performance indices are provided in the Data analysis section and Supplementary Methods.

### 2.2 Participants

Participants were adults who applied for the ICBT Wellbeing Course through the OTU. Referral sources included clinicians and other health system pathways as well as self-referral (e.g., online information, media, workplace/community sources). Applicants completed an online screening questionnaire assessing demographic and clinical characteristics, followed by a structured telephone interview with a clinician to confirm eligibility, clarify presenting problems, and assess risk. Individuals deemed ineligible were provided with information and referrals to alternative services. Eligible participants provided consent and were enrolled into the treatment platform prior to initiating the course. Routine-care eligibility criteria required applicants to be adults (≥18 years), reside in Saskatchewan, have sufficient internet access and ability to use the platform, and present with symptoms of depression and/or anxiety. Exclusion criteria generally included acute/high suicide risk or recent attempt, unmanaged psychosis or mania, and other clinical or contextual factors indicating that a different level or type of care was needed (e.g., severe substance-related impairment, significant cognitive impairment, or acute situational instability) or that would likely interfere with participating in an online course (e.g., inability to access the internet reliably or to engage with written materials).

### 2.3 Intervention

Participants received the transdiagnostic Wellbeing Course, a structured five-lesson ICBT program targeting depression and anxiety symptoms over approximately eight weeks (see Hadjistavropoulos et al., 2014; Titov et al., 2015). Lessons were delivered sequentially and included psychoeducation, skills practice, and homework assignments. Participants could also access additional self-help resources on the platform (e.g., sleep, worry, problem solving, motivation) and received therapist guidance primarily via asynchronous secure messaging with telephone support as needed, depending on the service model operating at the time.

### 2.4 Measures

Depression and anxiety symptom severity were assessed using the Patient Health Questionnaire–9 (PHQ-9; Kroenke et al., 2001) and the Generalized Anxiety Disorder–7 (GAD-7; Spitzer et al., 2006). Binary indicators of clinically elevated symptoms were created using standard thresholds (PHQ-9 score of 10 or higher; GAD-7 score of 10 or higher) and were used as baseline clinical predictors in the engagement models.

Demographic and contextual screening variables were obtained from routine intake questionnaires completed before treatment. These included age, gender, education, ethnicity, geographic location, relationship status, employment status, and current use of other mental health treatment. Engagement outcomes were derived from treatment-platform records of lesson completion.

### 2.5 Data analysis

All analyses were conducted in IBM SPSS Statistics (version 29). The analytic goal was to identify baseline predictors of engagement in routine-care ICBT using multivariable logistic regression. All predictors were derived from routine screening/intake (baseline) assessments completed before treatment started. Statistical tests were two-sided with α = 0.05.

Two binary engagement outcomes were examined: treatment completion (completed all five lessons vs fewer than five lessons; 1 = completer, 0 = non-completer) and early dropout (0–1 lesson vs ≥2 lessons; 1 = early dropout, 0 = engaged). Descriptive statistics were computed for analytic samples and stratified by engagement outcome where relevant. To provide context for temporal transportability, we also compared screening characteristics between the development period (TRAIN: 2013–2021) and temporal validation period (TEST: 2022–2024; Supplementary Table S2). This descriptive comparison was intended to characterize potential case-mix differences across service periods, rather than to test whether the periods were equivalent. Continuous variables are summarized using means and standard deviations, and categorical variables using frequencies and percentages.

Primary prediction models used complete-case data for the prespecified screening predictors. The final primary model sample included 9042 participants, including 6495 participants in TRAIN and 2547 participants in TEST. To describe engagement patterns over time, completion and early dropout rates were also summarized by screening year (Supplementary Fig. S1).

#### 2.5.1 Predictor sets and model specifications

Primary models used screening predictors available across the full study window (2013–2024): age in years; gender (women, men, other); education (high school or less, post-high school certificate/diploma, university degree); ethnicity (White, Indigenous, other); geographic location (small rural, small-to-medium city, large city); relationship status (single/never married/dating, married/common law, separated/divorced/widowed); employment status (paid work, homemaker/household, retired, student, unfit for work due to health, no paid work for other reasons); current mental health treatment (yes/no); clinically elevated depression (PHQ-9 ≥ 10); and clinically elevated anxiety (GAD-7 ≥ 10).

Separate multivariable logistic regression models were estimated for each engagement outcome: treatment completion and early dropout. All predictors were entered simultaneously. Categorical predictors were modeled using indicator coding with prespecified reference categories: women for gender, high school education or less for education, White for ethnicity, small rural location for geographic location, single/never married/dating for relationship status, paid work for employment status, no current mental health treatment, and non-clinical baseline status for PHQ-9 and GAD-7. As a secondary analysis, we also fitted a multinomial logistic regression using three mutually exclusive engagement categories: early dropout (0–1 lesson), partial engagement (2–4 lessons), and completion (5 lessons), with partial engagement as the reference category and the same baseline predictors as the primary models.

#### 2.5.2 Temporal validation and calibration

Temporal transportability was evaluated using the prespecified time-based split described in the Design section. For each outcome, logistic regression coefficients estimated in TRAIN were applied unchanged to participants in TEST to generate predicted probabilities. Performance was summarized using the Brier score for overall prediction error, area under the receiver operating characteristic curve (AUC) for discrimination, and calibration indices. Calibration was summarized using logistic recalibration, in which the observed outcome was regressed on the model linear predictor to estimate a calibration intercept and slope; ideal values are 0 for the intercept and 1 for the slope (Van Calster et al., 2019). Pooled models were also refitted in the full primary model sample to summarize adjusted associations and overall model performance across the full analytic period. As a sensitivity analysis addressing the change in assessment scheduling, we restricted analyses to the weekly-assessment era and fitted the same multivariable logistic regression models separately for participants screened in 2020–2021 and 2022–2024. Adjusted odds ratios and 95% confidence intervals were compared across periods for both treatment completion and early dropout. Additional details of scoring, performance metrics, calibration-by-bin summaries, and decision-rule evaluation are provided in the Supplementary Methods.

#### 2.5.3 Missing data

Missingness in this routine-care dataset reflected both routine nonresponse and changes in the routine-care assessment measures over time, with some baseline measures introduced later or not administered in all service years. Measures affected included lifetime mental-health diagnosis, treatment-waitlist status, and lifetime psychotropic-medication history. Primary prediction models were restricted to screening variables available across 2013–2024; consequently, complete-case loss for the primary models was minimal. Of 9057 participants with observed engagement outcomes, 9042 had complete data on all prespecified primary predictors and were included in the primary analyses.

## 3 Results

### 3.1 Sample characteristics and engagement patterns over time

Baseline demographic and clinical/psychosocial characteristics by lesson completion status are summarized in Table 1, Table 2. Full completers (five lessons) were older on average than partial completers, and the groups differed across several demographic characteristics (e.g., relationship status, ethnicity, education) and baseline clinical indicators, including depressive and anxiety symptom severity; however, most differences were small in magnitude (Table 1, Table 2).

**Table 1:** Demographic characteristics of participants at pre-treatment by lesson completion status.

Baseline characteristics	All Participants		Full completion (5 lessons)		Partial completion (<5 lessons)		p (X^2^, *t*-test)	*Effect size*
	N	M (SD)	*n*	M (SD)	*n*	M (SD)		
Age (range: 18–83)	9051	38.20 (13.42)	5863	39.95 (13.86)	3188	34.98 (11.94)	<0.001	*d* = 0.38

	n	%	n	%	n	%		
Gender								
Men	2275	25.1	1480	25.2	795	24.9	0.43	*V* = 0.01
Women	6674	73.7	4323	73.7	2351	73.7		
Other	107	1.2	63	1.1	44	1.4		
Marital status								
Single/never married/dating	2524	27.9	1462	24.9	1062	33.3	<0.001	*V* = 0.09
Married/common law	5586	61.7	3786	64.5	1800	56.4		
Separated/divorced/widowed	947	10.5	619	10.6	328	10.3		
Ethnicity								
Indigenous (First Nations, Inuit, Métis)	521	5.8	270	4.6	251	7.9	<0.001	*V* = 0.08
White	7864	86.9	5206	88.8	2658	83.3		
Other	667	7.4	387	6.6	280	8.8		
Education								
High school diploma or less	1876	20.7	1152	19.6	724	22.7	<0.001	*V* = 0.07
Post high school certificate/diploma	4023	44.4	2528	43.1	1495	46.9		
University education	3158	34.9	2187	37.3	971	30.4		
Employment								
Paid work	5411	59.8	3476	59.3	1935	60.7	<0.001	*V* = 0.11
Run the household	1090	12	694	11.8	396	12.4		
Retired	550	6.1	459	7.8	91	2.9		
Still at school	536	5.9	298	5.1	238	7.5		
Unfit for paid work (health)	785	8.7	529	9	256	8		
No paid work (other reasons)	684	7.6	410	7	274	8.6		
Location								
Small rural (population < 20*k*)	2920	32.2	1919	32.7	1001	31.4	0.36	*V* = 0.01
Small to medium city (20*k*–100*k*)	1538	17	980	16.7	558	17.5		
Large city (>100k)	4598	50.8	2967	50.6	1631	51.1		

Note. Full completers were defined as participants who completed all five lessons. Partial completers are those who completed fewer than five lessons. Ns vary across variables due to missing demographic data; percentages are calculated within each completion group using non-missing cases for that variable. Effect sizes are Cohen's d for age and Cramer's V for categorical variables.

**Table 2:** Clinical and psychosocial characteristics at pre-treatment by lesson completion status.

Baseline characteristics	All participants		Full completion (5 lessons)		Partial completion (<5 lessons)		P (X^2^, t-test)	*Effect size*
	N	M (SD)	n	M (SD)	n	M (SD)		
Generalized Anxiety Disorder – 7 items (GAD-7)	9057	11.69 (5.18)	5867	11.47 (5.18)	3190	12.09 (5.15)	<0.001	*d* = −0.12
Patient Health Questionnaire – 9 items (PHQ-9)	9057	11.86 (5.81)	5867	11.44 (5.81)	3190	12.65 (5.75)	<0.001	*d* = −0.21

	n	%	n	%	n	%		
Baseline GAD-7 score ≥ 10	5788	63.9	3667	62.5	2121	66.5	<0.001	φ = 0.04
Baseline PHQ-9 score ≥ 10	5720	63.2	3529	60.1	2191	68.7	<0.001	φ = 0.08
Living with someone								
Yes	7219	82.4	4669	82.1	2550	82.8	0.43	φ = 0.01
No	1547	17.6	1017	17.9	530	17.2		
Lifetime diagnosis								
Yes	3235	39.5	1951	37	1284	43.9	<0.001	φ = 0.07
No	4959	60.5	3320	63	1639	56.1		
Ever taken psychotropic medication (lifetime)								
Yes	6030	70.7	3770	68.9	2260	73.8	<0.001	φ = 0.05
No	2500	29.3	1698	31.1	802	26.2		
Taken psychotropic medication in last three months								
Yes	4937	56.3	3119	55	1818	58.8	<0.001	φ = 0.04
No	3831	43.7	2557	45	1274	41.2		
Currently receiving other mental health treatments								
Yes	4151	45.9	2694	45.9	1457	45.7	0.84	φ < 0.01
No	4900	54.1	3170	54.1	1730	54.3		

Note. Ns vary across variables due to routine-care missingness. Percentages are based on available cases within each variable. Effect sizes are Cohen's d for continuous variables and φ for binary categorical variables. Cohen's d was calculated as full completers minus partial completers.

Of the 9057 participants, 571 (6.3%) met the early-dropout definition (0–1 lesson), 2619 (28.9%) completed 2–4 lessons, and 5867 (64.8%) completed all five lessons. Compared with participants who completed at least two lessons, early dropouts were younger and showed small differences in several demographic and baseline clinical characteristics; most effect sizes were small, with the largest difference observed for age (*d* = −0.35; Supplementary Table S1).

To contextualize the temporal validation, screening characteristics were compared between the development period (TRAIN: 2013–2021) and temporal validation period (TEST: 2022–2024) in the primary model sample (Supplementary Table S2). The TEST period included participants who were slightly older on average and showed small differences in gender, ethnicity, geographic location, and employment status. Education, relationship status, current mental health treatment, and baseline symptom severity indicators showed trivial differences across periods. Overall, these comparisons suggest modest case-mix differences across service periods, primarily in demographic and geographic characteristics rather than baseline symptom severity.

Engagement outcomes also varied across screening years (Supplementary Fig. S1). Completion rates were highest in the earliest years, ranging from 75.0% in 2013 to 79.3% in 2014, then declined during 2015–2017, fluctuated around 71% during 2018–2019, decreased in 2020–2021, partially rebounded in 2022–2023, and declined again in 2024. Early dropout remained low overall but was generally higher in later years, ranging from 4.3% to 7.1% during 2013–2016 and from 5.9% to 8.9% during 2020–2024. Completion was higher in the lesson-anchored assessment era (2013–2019) than in the weekly assessment era (2020+; 70.9% vs 60.0%; Pearson χ^2^(1) = 116.99, *p* < .001), while early dropout was lower in the lesson-anchored era than in the weekly era (5.1% vs 7.2%; Pearson χ^2^(1) = 16.78, *p* < .001). These shifts in engagement rates provide important context for interpreting temporal calibration and transportability (Table 3).

**Table 3:** Performance and calibration indices for temporal validation and pooled models.

Outcome	Model/sample	N	Observed event rate	Mean predicted probability	Brier score	AUC	95% CI for AUC	Calibration intercept	Calibration slope
Completion	TRAIN	6495	0.657	0.657	0.216	0.624	0.610, 0.638	0.000	1.000
Completion	TEST	2547	0.624	0.669	0.223	0.643	0.622, 0.665	−0.250	1.060
Completion	Pooled	9042	0.648	0.649	0.217	0.630	0.618, 0.642	−0.003	0.997
Early dropout	TRAIN	6495	0.061	0.061	0.056	0.643	0.614, 0.671	0.000	1.000
Early dropout	TEST	2547	0.069	0.059	0.064	0.601	0.556, 0.645	−0.917	0.595
Early dropout	Pooled	9042	0.063	0.063	0.058	0.632	0.608, 0.655	−0.006	0.994

Note. TRAIN = model-development sample screened from 2013 to 2021; TEST = temporal validation sample screened from 2022 to 2024. Completion was coded 1 for completing all five core lessons and 0 otherwise. Early dropout was coded 1 for completing 0–1 lessons and 0 for completing 2–5 lessons. TRAIN performance estimates summarize model performance in the development sample. TEST performance estimates are based on predicted probabilities generated from models developed in TRAIN and applied without refitting to TEST. Pooled estimates are based on models refitted in the full primary model sample. Brier scores range from 0 to 1, with lower values indicating better overall prediction accuracy. AUC = area under the receiver operating characteristic curve. Calibration intercepts and slopes were estimated by regressing the observed outcome on the model linear predictor; ideal values are 0 for the intercept and 1 for the slope.

### 3.2 Temporal validation and model performance

Temporal validation using models developed in TRAIN (2013–2021) and applied without refitting to TEST (2022–2024), along with pooled model performance, is summarized in Table 3. For treatment completion, the TRAIN-derived score showed modest discrimination in TEST (AUC = 0.643, 95% CI [0.622, 0.665]) with a Brier score of 0.223. Mean predicted completion probability in TEST was 0.669, whereas observed completion was 0.624, indicating modest overprediction. Logistic recalibration produced a calibration intercept of −0.250 and a calibration slope of 1.060. The calibration slope was close to 1.0, while the AUC was similar in TRAIN (0.624) and TEST (0.643), indicating that discrimination was maintained over time.

For early dropout, the TRAIN-derived score showed weaker discrimination in TEST (AUC = 0.601, 95% CI [0.556, 0.645]) with a Brier score of 0.064. Because early dropout occurred in only 6.9% of the TEST sample, even a non-informative model assigning the same 6.9% risk to every participant would have a Brier score of approximately 0.064. The observed Brier score of 0.064 therefore indicates little improvement in overall probability accuracy from the baseline predictors. Mean predicted early-dropout probability in TEST was 0.059, whereas observed early dropout was 0.069, indicating slight underprediction. Logistic recalibration showed a calibration intercept of −0.917 and a calibration slope of 0.595, suggesting that the model tended to assign probabilities that were too high for some clients and too low for others in the later service period. Pooled models showed modest discrimination for both completion (AUC = 0.630, 95% CI [0.618, 0.642]) and early dropout (AUC = 0.632, 95% CI [0.608, 0.655]). Calibration-by-bin summaries and calibration plots for the temporal validation models are provided in Supplementary Tables S3–S4 and Supplementary Fig. S2A–B.

### 3.3 Extreme-group decision-rule utility

To evaluate whether the prediction scores could support simple service-planning decisions, we tested preplanned rules that flagged individuals in extreme predicted-risk groups in the 2022–2024 TEST sample (Table 4). For non-completion, flagging individuals in the lowest decile of predicted completion probability identified a subgroup with a non-completion rate of 61.8%, compared with 37.6% in the full TEST sample. This rule had high specificity (0.94) and moderate positive predictive value (0.62), but low sensitivity (0.16), indicating that most non-completers were not captured by the lowest-decile rule. Conversely, flagging individuals in the highest decile of predicted completion probability identified a subgroup with an 83.5% completion rate, with high specificity (0.96) and positive predictive value (0.84), but low sensitivity (0.13). For early dropout, flagging the highest-risk quintile identified a subgroup with an early-dropout rate of 11.6%, compared with 6.9% in the full TEST sample. This rule showed modest enrichment but limited positive predictive value (0.12), reflecting the low prevalence of early dropout.

**Table 4:** Extreme-group decision-rule utility in the temporal test set.

Outcome/rule	Evaluation sample	N	Prevalence	Flag rate	Sens	Spec	PPV	NPV
Non-completion: lowest completion decile	TEST 2022–2024	2547	0.38	0.10	0.16	0.94	0.62	0.65
Completion: highest completion decile	TEST 2022–2024	2547	0.62	0.10	0.13	0.96	0.84	0.40
Early dropout: highest-risk quintile	TEST 2022–2024	2547	0.07	0.20	0.34	0.81	0.12	0.94

Note. TEST = temporal validation sample screened from 2022 to 2024. Flag rate is the proportion flagged positive by the rule. Completion deciles were based on TRAIN-derived predicted probabilities for completion applied to the TEST sample. The lowest predicted completion decile was evaluated as a rule for identifying non-completion, and the highest predicted completion decile was evaluated as a rule for identifying completion. Early-dropout quintiles were based on TRAIN-derived predicted probabilities for early dropout applied to the TEST sample; the highest-risk quintile was evaluated as a rule for identifying early dropout. Sens = sensitivity; Spec = specificity; PPV = positive predictive value; NPV = negative predictive value. Because early dropout was uncommon, PPV values are expected to be modest even when the flagged group shows enrichment.

### 3.4 Baseline predictors of completion and early dropout

Adjusted associations from pooled complete-case multivariable logistic regression models are shown in Table 5, Table 6. In the pooled completion model (Table 5), older age was associated with higher odds of completing all five lessons (OR = 1.03 per year). Compared with participants with a high school education or less, participants with a university degree had higher odds of completion (OR = 1.32), whereas those with a post-high-school certificate or diploma did not differ significantly. Ethnicity was also associated with completion: Indigenous participants (OR = 0.62) and participants in the Other ethnicity category (OR = 0.76) had lower odds of completion than White participants. Participants who were separated/divorced/widowed had lower odds of completion than those who were single/never married/dating (OR = 0.78). Clinically elevated baseline depressive symptoms were associated with lower odds of completion (OR = 0.72), whereas clinically elevated baseline anxiety symptoms were not independently associated with completion. Gender, geographic location, employment status, and current mental health treatment were not independently associated with completion after adjustment.

**Table 5:** Pooled primary multivariable logistic regression predicting treatment completion.

Predictor (reference)	B	SE	OR	95% CI	p
Age, per year	0.028	0.002	1.028	[1.023, 1.033]	<0.001
Gender: men vs women	−0.052	0.054	0.950	[0.854, 1.055]	0.338
Gender: other vs women	0.010	0.204	1.010	[0.677, 1.506]	0.962
Education: post-high school vs high school or less	0.043	0.060	1.044	[0.928, 1.174]	0.472
Education: university degree vs high school or less	0.280	0.065	1.323	[1.165, 1.504]	<0.001
Ethnicity: Indigenous vs White	−0.472	0.093	0.624	[0.519, 0.748]	<0.001
Ethnicity: other vs White	−0.273	0.085	0.761	[0.644, 0.899]	0.001
Relationship: married/common law vs single/never married/dating	0.078	0.057	1.081	[0.967, 1.208]	0.169
Relationship: separated/divorced/widowed vs single/never married/dating	−0.249	0.090	0.780	[0.653, 0.931]	0.006
Employment: homemaker/household vs paid work	−0.041	0.073	0.960	[0.832, 1.107]	0.571
Employment: retired vs paid work	0.264	0.136	1.302	[0.998, 1.699]	0.052
Employment: student vs paid work	0.046	0.098	1.047	[0.864, 1.269]	0.641
Employment: unfit for work due to health vs paid work	0.096	0.086	1.101	[0.931, 1.302]	0.261
Employment: no paid work for other reasons vs paid work	−0.026	0.086	0.975	[0.823, 1.155]	0.768
Location: small-to-medium city vs small rural	0.018	0.068	1.018	[0.891, 1.164]	0.788
Location: large city vs small rural	−0.017	0.053	0.983	[0.887, 1.090]	0.747
Current mental health treatment: yes vs no	0.008	0.046	1.008	[0.921, 1.104]	0.860
PHQ-9 clinical baseline: yes vs no	−0.330	0.053	0.719	[0.648, 0.798]	<0.001
GAD-7 clinical baseline: yes vs no	0.083	0.053	1.087	[0.980, 1.205]	0.114

Note. Model estimated in the pooled primary complete-case sample (*N* = 9042). The dependent variable was treatment completion (1 = completed all five lessons; 0 = did not complete all five lessons). Values are regression coefficients (B), standard errors (SE), odds ratios (OR = Exp[B]), and 95% confidence intervals (CI). Odds ratios greater than 1 indicate higher odds of completion. Reference categories were: women for gender, high school education or less for education, White for ethnicity, single/never married/dating for relationship status, paid work for employment status, small rural location for geographic location, no current mental health treatment, and non-clinical baseline status on the PHQ-9 and GAD-7.

**Table 6:** Pooled primary multivariable logistic regression predicting early dropout.

Predictor (reference)	B	SE	OR	95% CI	p
Age, per year	−0.027	0.005	0.974	[0.964, 0.983]	<0.001
Gender: men vs women	0.066	0.103	1.068	[0.872, 1.308]	0.525
Gender: other vs women	−0.434	0.427	0.648	[0.280, 1.497]	0.310
Education: post-high school vs high school or less	−0.062	0.110	0.940	[0.757, 1.166]	0.571
Education: university degree vs high school or less	−0.371	0.127	0.690	[0.538, 0.885]	0.003
Ethnicity: Indigenous vs White	0.531	0.153	1.700	[1.260, 2.294]	<0.001
Ethnicity: other vs White	0.428	0.147	1.534	[1.151, 2.045]	0.004
Relationship: married/common law vs single/never married/dating	−0.146	0.106	0.864	[0.702, 1.063]	0.168
Relationship: separated/divorced/widowed vs single/never married/dating	−0.054	0.182	0.947	[0.663, 1.354]	0.766
Employment: homemaker/household vs paid work	0.004	0.144	1.004	[0.758, 1.330]	0.977
Employment: retired vs paid work	0.229	0.278	1.258	[0.730, 2.168]	0.409
Employment: student vs paid work	−0.047	0.171	0.954	[0.682, 1.334]	0.783
Employment: unfit for work due to health vs paid work	−0.098	0.172	0.907	[0.647, 1.271]	0.570
Employment: no paid work for other reasons vs paid work	0.102	0.155	1.107	[0.817, 1.501]	0.512
Location: small-to-medium city vs small rural	0.036	0.131	1.036	[0.802, 1.340]	0.784
Location: large city vs small rural	0.061	0.102	1.063	[0.869, 1.299]	0.554
Current mental health treatment: yes vs no	−0.003	0.089	0.997	[0.836, 1.187]	0.969
PHQ-9 clinical baseline: yes vs no	0.164	0.105	1.179	[0.959, 1.448]	0.118
GAD-7 clinical baseline: yes vs no	0.039	0.104	1.040	[0.848, 1.276]	0.705

Note. Model estimated in the pooled primary complete-case sample (*N* = 9042). The dependent variable was early dropout (1 = completed 0–1 lessons; 0 = completed two or more lessons). Values are regression coefficients (B), standard errors (SE), odds ratios (OR = Exp[B]), and 95% confidence intervals (CI). Odds ratios greater than 1 indicate higher odds of early dropout. Reference categories were: women for gender, high school education or less for education, White for ethnicity, single/never married/dating for relationship status, paid work for employment status, small rural location for geographic location, no current mental health treatment, and non-clinical baseline status on the PHQ-9 and GAD-7.

In the pooled early-dropout model (Table 6), older age was associated with lower odds of early dropout (OR = 0.97 per year), meaning that younger participants had higher odds of early dropout. Compared with participants with a high school education or less, participants with a university degree had lower odds of early dropout (OR = 0.69), whereas those with a post-high-school certificate or diploma did not differ significantly. Indigenous participants (OR = 1.70) and participants in the Other ethnicity category (OR = 1.53) had higher odds of early dropout than White participants. Gender, relationship status, employment status, geographic location, current mental health treatment, clinically elevated baseline depressive symptoms, and clinically elevated baseline anxiety symptoms were not independently associated with early dropout after adjustment.

In a secondary multinomial analysis using partial engagement (2–4 lessons) as the reference category, the overall model was significant, χ^2^(38) = 469.12, *p* < .001, Nagelkerke *R*^2^ = 0.063. Predictors of completion versus partial engagement were generally consistent with the primary completion model. In contrast, none of the baseline predictors significantly distinguished early dropout from partial engagement, although several estimates were in the same direction as those in the primary early-dropout model (Supplementary Table S5).

In the weekly-assessment-era sensitivity analysis, adjusted predictor-outcome associations were broadly similar across 2020–2021 and 2022–2024, although some period-specific differences were observed (Supplementary Table S6). For completion, the age association was nearly identical across periods (OR = 1.034 vs 1.030), while associations for university education, Indigenous ethnicity, and elevated PHQ-9 symptoms were in the same direction but somewhat stronger in 2022–2024. For early dropout, age showed an identical association across periods (OR = 0.967 in both), and estimates for university education and elevated PHQ-9 symptoms were also similar. Greater variation was observed for Indigenous ethnicity and geographic location.

## 4 Discussion

This study examined screening predictors of engagement in a large routine-care ICBT service using multivariable logistic regression and a prespecified earlier-years to later-years temporal validation. Engagement varied across service years, with completion generally lower and early dropout generally higher in later years than in the earliest years of service delivery. Prediction performance was modest overall. In TEST, the completion model showed modest ability to distinguish completers from non-completers and, on average, predicted a somewhat higher completion rate than was observed. The early-dropout model showed weaker ability to distinguish early dropouts from other participants and, on average, predicted a slightly lower early-dropout rate than was observed. In models fitted to the full analytic sample while accounting for all baseline predictors, engagement was more consistently associated with demographic factors than with symptom severity at screening. Completion was more likely among older clients, those with a university degree, White participants, and those who were not separated/divorced/widowed. Early dropout was more likely among younger clients, those without a university degree, Indigenous participants, and participants in the other ethnicity category.

### 4.1 Engagement as a staged process

A central implication is that “attrition” is not a single phenomenon, and this distinction is important in routine-care ICBT because early discontinuation and later non-completion may have different predictors and service implications. Our staged engagement outcomes are consistent with broader CBT evidence showing that attrition occurs at multiple points, including before treatment starts and during treatment, and that dropout varies by delivery format and clinical context (Fernandez et al., 2015). Internet-delivered interventions, including ICBT, often lose users at multiple points (non-initiation, early discontinuation, and later non-completion), and treating engagement as a single “dropout” endpoint can blur distinct processes with different determinants and service implications (Christensen and Mackinnon, 2006; Eysenbach, 2005; Linardon et al., 2025; Sieverink et al., 2017). Recent engagement scholarship also emphasizes that engagement is multidimensional (behavioral, cognitive, affective) and dynamic over time rather than a one-time outcome (Nahum-Shani et al., 2022). Although the present study operationalized engagement using behavioral indicators (lesson completion and early dropout), the staged endpoints yielded meaningfully different predictability and partially different predictor patterns. The secondary three-category analysis showed a similar pattern: baseline characteristics were more informative for distinguishing completion from partial engagement than for distinguishing early dropout from partial engagement. Early disengagement may reflect not only short-term barriers and early experiences that are difficult to capture at screening, but also external factors such as life events, competing demands, or stopping after sufficient improvement (Lawler et al., 2021). Thus, distinguishing engagement stages was informative for showing that completion and early dropout were not equally predictable. Baseline variables modestly predicted completion but did not reliably identify individuals at risk of early dropout.

The results align with evidence from both meta-analytic work on online anxiety interventions and national digital mental health services showing substantial variability in uptake and completion (Bisby et al., 2022; Cross et al., 2022). In the present study, predictors differed across completion and early dropout, and baseline models were modest for completion but weaker and less well calibrated for early dropout when applied to later service years.

These findings reinforce the value of examining engagement stages separately, as in prior work distinguishing treatment initiation from subsequent continuation or dropout (Rotondi et al., 2024; Wu et al., 2022). From a service planning perspective, these findings suggest that screening-based risk stratification may have limited usefulness for identifying early dropout on its own, and that engagement support approaches may need to incorporate early in-treatment indicators rather than relying only on screening variables.

The pooled models suggest that engagement in routine-care ICBT was more consistently associated with demographic characteristics than with baseline symptom-severity indicators. Older age and university education were associated with higher odds of completion and lower odds of early dropout, whereas Indigenous participants and participants in the other ethnicity category had lower odds of completion and higher odds of early dropout than White participants. Participants who were separated/divorced/widowed also had lower odds of completion, although relationship status was not independently associated with early dropout. Clinically elevated depressive symptoms at baseline were associated with lower odds of completion, but not with early dropout, and clinically elevated anxiety symptoms were not independently associated with either outcome. These findings suggest that baseline symptom severity alone is unlikely to provide a sufficient basis for identifying clients at risk of disengagement, particularly early dropout.

Two additional considerations may help explain why symptom severity at screening was a weaker predictor of engagement than demographic characteristics. First, engagement often follows different usage patterns over time, such as intermittent start-stop participation versus continuous participation, so a single “non-completer” group can include people who stop for different reasons; screening predictors may not distinguish these pathways well (Chien et al., 2020). Second, qualitative studies of ICBT discontinuation commonly point to practical barriers, such as time constraints and competing demands, shifts in motivation, and early experiences with the program or support as reasons for stopping, factors that are not well captured by symptom severity at screening (Koelen et al., 2025; Lawler et al., 2021). Similar reasons are also reported in face-to-face psychotherapy dropout research (Fernandez et al., 2015; Kullgard et al., 2022; Swift and Greenberg, 2012).

### 4.2 Temporal transportability in long-running routine-care programs

An important contribution of this study is the explicit focus on temporal transportability, that is, whether models developed in earlier service years retain useful performance when applied, without refitting, to later service years. Because routine-care digital services evolve over time, prediction performance should be evaluated in later service years and with attention to calibration as well as discrimination (Bennemann et al., 2022; Collins et al., 2024). Consistent with expected changes over time, transportability differed by outcome. The completion model retained modest discrimination in TEST, while its average predicted completion rate was somewhat higher than the rate actually observed, suggesting that the main change was a lower overall completion rate in TEST rather than poorer discrimination. The early-dropout model showed weaker discrimination, slightly underestimated the overall early-dropout rate, and had a lower calibration slope, indicating that the spread of predicted risks was less well calibrated in later service years. The early-dropout Brier score was also essentially the same as that of a simple model based only on the overall early-dropout rate, indicating that the baseline predictors added little to overall probability accuracy. This pattern shows why temporal validation is necessary for service-related prediction in long-running routine-care programs. Different aspects of model performance may change over time; in this study, discrimination was relatively stable for completion, whereas early-dropout prediction was less stable in the later service period. Engagement patterns in the OTU service shifted substantially over time, with completion decreasing and early dropout increasing in later years. These changes in outcome rates likely contributed to the miscalibration observed when earlier-year models were applied to 2022–2024, because models trained in earlier years implicitly expected different baseline completion and dropout rates than those observed later. The TRAIN/TEST screening comparison also suggests that temporal validation occurred in the context of modest participant case-mix differences: the later evaluation period included slightly older participants and small shifts in gender, ethnicity, geographic location, and employment status, while education, relationship status, current mental health treatment, and baseline PHQ-9 and GAD-7 severity showed trivial differences. These differences may help contextualize calibration drift, although they should not be interpreted as causal explanations for changes in model performance.

In this service, a major operational change occurred in assessment administration, with lesson-anchored symptom monitoring during 2013–2019 and weekly monitoring from 2020 onward. However, a sensitivity analysis restricted to the weekly-assessment era showed that many adjusted predictor-outcome associations were broadly similar between 2020–2021 and 2022–2024, although some predictor-specific differences remained. Routine-care services may also evolve in other ways over time, including enrollment and onboarding procedures, staffing and workflow, platform features, messaging practices, and client referral pathways. More broadly, the rapid growth of virtual care, including physician-based mental health services delivered by phone or video in Canada and Saskatchewan during and after the COVID-19 period, may also have influenced demand, referral pathways, and the mix of clients entering ICBT in later years (Canadian Institute for Health Information, 2022). Together, these implementation-era and system-level shifts provide plausible context for the change in the overall completion rate and the weaker early-dropout prediction observed in 2022–2024. These trends are best viewed as indicators of a changing service context rather than evidence of any single driver. COVID-19 era system changes and routine program evolution may both have contributed. Declining completion should also be interpreted cautiously because some non-completion may reflect stopping after meaningful early improvement.

### 4.3 Implications

The findings support distinguishing stages of engagement but provide limited support for using baseline variables to identify individuals at risk of early dropout. This distinction is consistent with recent personalization frameworks that differentiate baseline risk assessment from responsiveness during treatment (Aafjes-van Doorn and Christensen, 2026). Future research should examine whether engagement support after the early phase is more useful when informed by early clinical response and the client's felt need to continue. Unlike in randomized trials, where completion is often treated as the primary adherence target, routine-care services do not necessarily aim for full completion for every client; some people stop early because they improve quickly or feel they have received enough benefit.

In this service, demographic factors (e.g., age, education, ethnicity, and relationship status) were more consistently linked to engagement than symptom severity. Given the limited predictive value of baseline variables for early dropout, these characteristics should not be used alone to determine who receives closer early monitoring. This caution is especially important for ethnicity. Differences in engagement by ethnicity may reflect structural, contextual, or service-related barriers rather than intrinsic individual risk, and using ethnicity alone to allocate additional monitoring or support could reinforce inequities. The ‘Other ethnicity’ category was also heterogeneous, and the Indigenous subgroup was comparatively small, so these estimates should be interpreted cautiously. Early in-treatment signals, such as lesson progress, time between log-ins, therapist contact, and emerging barriers to engagement, may be more informative and warrant evaluation in future research. Future studies could evaluate whether brief targeted telephone check-ins improve engagement for clients showing early signs of disengagement. Evidence from guided digital care suggests that additional contact can improve adherence in some settings (Pihlaja et al., 2020), although the value of targeting such support to clients at risk of early dropout remains to be established.

After the initial engagement period (e.g., after Dose 2), future research should examine whether support informed by early clinical response and engagement patterns improves retention. Because prior research links early engagement indicators to later discontinuation risk (Wu et al., 2022), services may benefit from monitoring engagement and clinical change, although the value of using these indicators to guide additional support requires further evaluation. Future studies should examine whether early symptom response and early engagement indicators improve prediction of subsequent completion and disengagement beyond baseline characteristics. For the clients who improve early, future research should examine how symptom change can inform decisions about whether additional lessons are still needed. For clients who are not improving or are struggling to engage, future research could examine whether stepped support addressing barriers, treatment fit, pacing, reminders, or support intensity improves engagement, given broader evidence linking greater engagement with better outcomes in digital interventions (Gan et al., 2021). Finally, engagement should be interpreted in context: more use is not automatically better, and services benefit from defining what “intended engagement” means for their program and evaluating engagement alongside outcomes (Smith et al., 2025).

### 4.4 Limitations

This study used observational routine-care data, so associations should not be interpreted causally. Unmeasured factors, such as digital literacy, competing demands, preferences, therapist factors, and early treatment experiences, likely influence engagement (Koelen et al., 2025; Lawler et al., 2021). The primary models were also restricted to baseline screening variables available across the primary analytic period. This approach supported temporal validation across service years, but it meant that potentially important early in-treatment indicators, such as initial logins, lesson access, early therapist contact, and first-week program experiences, were not included. This limitation is particularly relevant for early dropout, which may depend more strongly on what happens immediately after enrollment than on screening characteristics alone. Early dropout was also relatively uncommon, occurring in approximately 6–7% of participants, which limited the number of events available for model estimation and temporal validation and increased uncertainty around performance estimates. The service also evolved over time through both operational changes and intentional refinements, including the shift in assessment administration and other adjustments in guidance and course delivery procedures, such as contact schedules, review of homework records, extensions, booster resources, and related workflow updates. Because these changes can alter engagement rates and the relationships between predictors and outcomes, ongoing monitoring and recalibration would be necessary if engagement prediction models were implemented in routine service delivery. Finally, lesson-based outcomes, including completion and early dropout, are clinically meaningful but imperfect proxies: some non-completers may stop after experiencing sufficient benefit, and some completers may persist despite limited gains. More broadly, engagement research cautions against equating “more clicks” or “more modules” with meaningful engagement without considering intended use and clinical context (Nahum-Shani et al., 2022; Sieverink et al., 2017). Despite these limitations, the large routine-care sample and prespecified temporal validation provide an implementation-relevant picture of engagement patterns and their screening correlates in a long-running ICBT service.

### 4.5 Conclusions

Engagement in routine-care ICBT was more consistently related to demographic characteristics, including age, education, ethnicity, and relationship status, than to symptom severity at screening. Early dropout was especially difficult to predict from screening variables alone. Temporal validation showed that discrimination for completion was maintained in later service years, whereas discrimination for early dropout was weaker. Completion was modestly overpredicted in TEST, whereas early dropout showed slight underprediction and weaker separation of higher- and lower-risk clients, underscoring the importance of evaluating prediction models in later service periods and monitoring calibration over time. Practically, baseline variables had limited usefulness for identifying individuals at risk of early dropout. Future research should test whether targeted supports informed by early indicators, such as lesson progress, symptom change, or therapist contact, improve retention and equity in routine care.

## Ethics declaration

### Informed consent and patient details

Written informed consent to take part in the study and to publish the article has been obtained from all participants or their legal representatives. The privacy rights of participants have been observed.

### Studies in human

This study was performed in compliance with relevant laws, regulatory frameworks and guidelines where the research took place.

Ethics committee approval was not required under relevant laws and institutional guidelines. Secondary analysis of the trial data.

## Declaration of Generative AI and AI-assisted technologies in the writing process

During the preparation of this work, RPS used ChatGPT (OpenAI) to improve grammar and phrasing and to generate SPSS syntax and equations presented in the Supplementary Methods. After using this tool, the authors reviewed and edited the content as needed and take full responsibility for the content of the publication.

## Funding

The Online Therapy Unit is funded by the Saskatchewan Ministry of Health. No funding was received specifically for this study.

## Declaration of competing interest

The authors declare no conflict of interest.

## Data Availability

The data used in this study were collected as part of routine care and have not been deposited in a repository because data sharing through a public repository was not included in the patient consent process. Data may be available from the corresponding author upon reasonable request, subject to applicable ethics, privacy, and data-sharing requirements.

## References

[bb0005] Aafjes-van Doorn K., Christensen H. (2026). A clinically actionable framework for personalizing iCBT to improve depression outcomes. NPJ Digit. Med..

[bb0010] Bennemann B., Schwartz B., Giesemann J., Lutz W. (2022). Predicting patients who will drop out of out-patient psychotherapy using machine learning algorithms. Br. J. Psychiatry.

[bb0015] Bisby M.A., Karin E., Hathway T., Scott A.J., Heriseanu A.I., Dudeney J., Dear B.F. (2022). A meta-analytic review of randomized clinical trials of online treatments for anxiety: inclusion/exclusion criteria, uptake, adherence, dropout, and clinical outcomes. J. Anxiety Disord..

[bb0020] Canadian Institute for Health Information (2022). Virtual care: use of physician mental health services in Canada. https://www.cihi.ca/en/virtual-care-use-of-physician-mental-health-services-in-canada.

[bb0025] Chien I., Enrique A., Palacios J., Regan T., Keegan D., Carter D., Belgrave D. (2020). A machine learning approach to understanding patterns of engagement with internet-delivered mental health interventions. JAMA Netw. Open.

[bb0030] Christensen H., Mackinnon A. (2006). The law of attrition revisited. J. Med. Internet Res..

[bb0035] Collins G.S., Dhiman P., Ma J., Schlussel M.M., Archer L., Van Calster B., Harrell F.E., Martin G.P., Moons K.G.M., van Smeden M., Sperrin M., Bullock G.S., Riley R.D. (2024). Evaluation of clinical prediction models (part 1): from development to meaningful evaluation. BMJ.

[bb0040] Cross S.P., Karin E., Staples L.G., Bisby M.A., Ryan K., Duke G., Titov N. (2022). Factors associated with treatment uptake, completion, and subsequent symptom improvement in a national digital mental health service. Internet Interv..

[bb0045] Cuijpers P., Harrer M., Miguel C., Ciharova M., Papola D., Basic D., Botella C., Cristea I., de Ponti N., Donker T. (2025). Cognitive behavior therapy for mental disorders in adults: a unified series of meta-analyses. JAMA Psychiatry.

[bb0050] Edmonds M., Hadjistavropoulos H., Schneider L., Dear B., Titov N. (2018). Who benefits most from therapist-assisted internet-delivered cognitive behaviour therapy in clinical practice? Predictors of symptom change and dropout. J. Anxiety Disord..

[bb0055] Etzelmueller A., Vis C., Karyotaki E., Baumeister H., Titov N., Berking M., Cuijpers P., Riper H., Ebert D.D. (2020). Effects of internet-based cognitive behavioral therapy in routine care for adults in treatment for depression and anxiety: systematic review and meta-analysis. J. Med. Internet Res..

[bb0060] Eysenbach G. (2005). The law of attrition. J. Med. Internet Res..

[bb0065] Fernandez E., Salem D., Swift J.K., Ramtahal N. (2015). Meta-analysis of dropout from cognitive behavioral therapy: magnitude, timing, and moderators. J. Consult. Clin. Psychol..

[bb0070] Gan D.Z., McGillivray L., Han J., Christensen H., Torok M. (2021). Effect of engagement with digital interventions on mental health outcomes: a systematic review and meta-analysis. Front. Digit. Health.

[bb0075] Hadjistavropoulos H., Peynenburg V. (2025). Fifteen years of internet-delivered cognitive behaviour therapy in Saskatchewan: expanding access to evidence-based care. Healthc. Q..

[bb0085] Hadjistavropoulos H., Peynenburg V., Thiessen D.L., Nugent M., Karin E., Staples L., Dear B.F., Titov N. (2022). Utilization, patient characteristics, and longitudinal improvements among patients from a provincially funded transdiagnostic internet-delivered cognitive behavioural therapy program: observational study of trends over 6 years. Can. J. Psychiatr..

[bb0080] Hadjistavropoulos H.D., Alberts N.M., Nugent M., Marchildon G. (2014). Improving access to psychological services through therapist-assisted, Internet-delivered cognitive behaviour therapy. Can. Psychol..

[bb0090] Hedman-Lagerlöf E., Carlbring P., Svärdman F., Riper H., Cuijpers P., Andersson G. (2023). Therapist-supported internet-based cognitive behaviour therapy yields similar effects as face-to-face therapy for psychiatric and somatic disorders: an updated systematic review and meta-analysis. World Psychiatry.

[bb0095] Koelen J.A., de Koning L., Nottage M.K., Klein A.M., van der Heijde C.M., Vonk P., Wiers R.W. (2025). Dropout and completion in iCBT for university students: insights from a thematic analysis. Internet Interv..

[bb0100] Kroenke K., Spitzer R.L., Williams J.B. (2001). The PHQ-9: validity of a brief depression severity measure. J. Gen. Intern. Med..

[bb0105] Kullgard N., Holmqvist R., Andersson G. (2022). Premature dropout from psychotherapy: prevalence, perceived reasons and consequences as rated by clinicians. Clin. Psychol. Eur..

[bb0110] Lawler K., Earley C., Timulak L., Enrique A., Richards D. (2021). Dropout from an internet-delivered cognitive behavioral therapy intervention for adults with depression and anxiety: qualitative study. JMIR Form. Res..

[bb0115] Linardon J., Messer M., Reid R., Bolger T., Andersson G. (2025). Absolute and relative rates of treatment non-initiation, dropout, and attrition in internet-based and face-to-face cognitive-behavioral therapy: a meta-analysis of randomized controlled trials. Cogn. Behav. Ther..

[bb0120] McCall H.C., Hadjistavropoulos H.D., Sundström C.R.F. (2021). Exploring the role of persuasive design in unguided internet-delivered cognitive behavioral therapy for depression and anxiety among adults: systematic review, meta-analysis, and meta-regression. J. Med. Internet Res..

[bb0125] Nahum-Shani I., Shaw S.D., Carpenter S.M., Murphy S.A., Yoon C. (2022). Engagement in digital interventions. Am. Psychol..

[bb0130] Österman S., Axelsson E., Forsell E., Svanborg C., Lindefors N., Hedman-Lagerlöf E., Ivanov V.Z. (2024). Effectiveness and prediction of treatment adherence to guided internet-based cognitive behavioral therapy for health anxiety: a cohort study in routine psychiatric care. Internet Interv..

[bb0135] Peynenburg V., Wilhelms A., Sapkota R., Nugent M., Owens K., Titov N., Dear B., Hadjistavropoulos H. (2022). Understanding client difficulties in transdiagnostic internet-delivered cognitive behaviour therapy: a qualitative analysis of homework reflections. J. Clin. Med..

[bb0140] Pihlaja S., Lahti J., Lipsanen J.O., Ritola V., Gummerus E.-M., Stenberg J.-H., Joffe G. (2020). Scheduled telephone support for internet cognitive behavioral therapy for depression in patients at risk for dropout: pragmatic randomized controlled trial. J. Med. Internet Res..

[bb0145] Roemmel N., Bahmane S., Hadjistavropoulos H.D., Nugent M., Lieb R., Meinlschmidt G. (2025). Prediction of treatment outcome in patients receiving internet-delivered cognitive behavioural therapy for depressive and anxiety symptoms: a machine learning analysis of data from a healthcare-embedded longitudinal study. BMJ Open.

[bb0150] Rotondi A.J., Herbeck Belnap B., Rothenberger S., Feldman R., Hanusa B., Rollman B.L. (2024). Predictors of use and drop out from a web-based cognitive behavioral therapy program and health community for depression and anxiety in primary care patients: secondary analysis of a randomized controlled trial. JMIR Ment. Health.

[bb0155] Sieverink F., Kelders S.M., van Gemert-Pijnen J.E. (2017). Clarifying the concept of adherence to eHealth technology: systematic review on when usage becomes adherence. J. Med. Internet Res..

[bb0160] Smith K.A., Ward T., Lambe S., Ostinelli E.G., Blease C., Gant T., Cipriani A., Freeman D. (2025). Engagement and attrition in digital mental health: current challenges and potential solutions. NPJ Digit. Med..

[bb0165] Spitzer R.L., Kroenke K., Williams J.B., Löwe B. (2006). A brief measure for assessing generalized anxiety disorder: the GAD-7. Arch. Intern. Med..

[bb0170] Swift J.K., Greenberg R.P. (2012). Premature discontinuation in adult psychotherapy: a meta-analysis. J. Consult. Clin. Psychol..

[bb0175] Titov N., Dear B.F., Staples L.G., Bennett-Levy J., Klein B., Rapee R.M., Shann C., Richards D., Andersson G., Ritterband L. (2015). MindSpot clinic: an accessible, efficient, and effective online treatment service for anxiety and depression. Psychiatr. Serv..

[bb0180] Van Calster B., McLernon D.J., van Smeden M., Wynants L., Steyerberg E.W. (2019). Calibration: the Achilles heel of predictive analytics. BMC Med..

[bb0185] Wu M.S., Chen S.-Y., Wickham R.E., Leykin Y., Varra A., Chen C., Lungu A. (2022). Predicting non-initiation of care and dropout in a blended care cognitive behavioral therapy intervention. Digit. Health.

[bb0190] Zantvoort K., Hentati Isacsson N., Funk B., Kaldo V. (2024). Dataset size versus homogeneity: a machine learning study on pooling intervention data in e-mental health dropout predictions. Digit. Health.

