# Supplementary material for: Predicting completion and early dropout in routine-care internet-delivered cognitive behaviour therapy: A temporal validation study
Source: Internet Interv. 2026 Oct 12;46:101011. doi: 10.1016/j.invent.2026.101011 (PMC13682379; doi:10.1016/j.invent.2026.101011)
Supplement: Supplementary file 1 [file mmc1.docx]

**Supplementary Figures**

**Figure S1.**

Engagement outcomes by screening year (2013–2024).

Percentage

| Screening year | Screening year | Completion rate (%) | Early dropout rate (%) |
| --- | --- | --- | --- |
| 2013 | 2013 | 75 | 7.1 |
| 2014 | 2014 | 79.3 | 4.3 |
| 2015 | 2015 | 70.3 | 4.4 |
| 2016 | 2016 | 70.6 | 6.3 |
| 2017 | 2017 | 66.3 | 5.6 |
| 2018 | 2018 | 71.1 | 4.9 |
| 2019 | 2019 | 71.8 | 4.8 |
| 2020 | 2020 | 59.9 | 6.6 |
| 2021 | 2021 | 55.3 | 8.5 |
| 2022 | 2022 | 64.8 | 6.2 |
| 2023 | 2023 | 67.2 | 5.9 |
| 2024 | 2024 | 54.2 | 8.9 |

Note. Completion was defined as completing all five lessons. Early dropout was defined as completing 0–1 lesson.

**Figure S2A.**

Calibration plot for the completion model in the TEST sample.

Observed probability

| Predicted probability | Observed completion rate |
| --- | --- |
| 0.483 | 0.382 |
| 0.553 | 0.535 |
| 0.593 | 0.547 |
| 0.626 | 0.578 |
| 0.657 | 0.599 |
| 0.684 | 0.665 |
| 0.712 | 0.641 |
| 0.745 | 0.69 |
| 0.787 | 0.765 |
| 0.85 | 0.835 |

| Predicted probability | Perfect calibration |
| --- | --- |
| 0.3 | 0.3 |
| 0.9 | 0.9 |

Note. Points represent deciles of TRAIN-derived predicted completion probability. The diagonal line represents perfect calibration.

**Figure S2B.**

Calibration plot for the early-dropout model in the TEST sample.

Observed probability

| Predicted probability | Observed early-dropout rate |
| --- | --- |
| 0.025 | 0.045 |
| 0.039 | 0.053 |
| 0.052 | 0.055 |
| 0.068 | 0.077 |
| 0.11 | 0.116 |

| Predicted probability | Perfect calibration |
| --- | --- |
| 0 | 0 |
| 0.15 | 0.15 |

Note. Points represent quintiles of TRAIN-derived predicted early-dropout probability. The diagonal line represents perfect calibration.
