# Supplementary material for: Predicting completion and early dropout in routine-care internet-delivered cognitive behaviour therapy: A temporal validation study
Source: Internet Interv. 2026 Oct 12;46:101011. doi: 10.1016/j.invent.2026.101011 (PMC13682379; doi:10.1016/j.invent.2026.101011)
Supplement: Supplementary file 2 [file mmc2.docx]

**Supplementary Tables**

**Table S1.**
Baseline Demographic, Clinical, and Psychosocial Characteristics by Early-Dropout Status

| Baseline characteristic | Engaged ≥2 lessons, n (%) | Early dropout 0–1 lesson, n (%) | *p* | Effect size |
| --- | --- | --- | --- | --- |
| Age, M (SD) | 38.49 (13.47) | 33.82 (11.97) | < .001 | d = −0.35 |
| Gender |  |  | 0.904 | V = .01 |
| Women | 6256 (73.7) | 418 (73.2) |  |  |
| Men | 2128 (25.1) | 147 (25.7) |  |  |
| Other | 101 (1.2) | 6 (1.1) |  |  |
| Marital status |  |  | < .001 | V = .06 |
| Single/never married/dating | 2307 (27.2) | 217 (38.0) |  |  |
| Married/common law | 5280 (62.2) | 306 (53.6) |  |  |
| Separated/divorced/widowed | 899 (10.6) | 48 (8.4) |  |  |
| Ethnicity |  |  | < .001 | V = .06 |
| White | 7409 (87.4) | 455 (79.7) |  |  |
| Indigenous | 466 (5.5) | 55 (9.6) |  |  |
| Other | 606 (7.1) | 61 (10.7) |  |  |
| Education |  |  | < .001 | V = .04 |
| High school diploma or less | 1734 (20.4) | 142 (24.9) |  |  |
| Post-high school certificate/diploma | 3748 (44.2) | 275 (48.2) |  |  |
| University degree | 3004 (35.4) | 154 (27.0) |  |  |
| Employment |  |  | 0.001 | V = .05 |
| Paid work | 5074 (59.8) | 337 (59.0) |  |  |
| Homemaker/household | 1023 (12.1) | 67 (11.7) |  |  |
| Retired | 531 (6.3) | 19 (3.3) |  |  |
| Student | 487 (5.7) | 49 (8.6) |  |  |
| Unfit for work due to health | 742 (8.7) | 43 (7.5) |  |  |
| No paid work for other reasons | 628 (7.4) | 56 (9.8) |  |  |
| Location |  |  | 0.454 | V = .01 |
| Small rural | 2749 (32.4) | 171 (29.9) |  |  |
| Small-to-medium city | 1435 (16.9) | 103 (18.0) |  |  |
| Large city | 4301 (50.7) | 297 (52.0) |  |  |
| Generalized Anxiety Disorder –7 items (GAD-7) | 11.65 (5.18) | 12.26 (5.19) | .006 | d = 0.12 |
| Patient Health Questionnaire – 9 items (PHQ-9) | 11.79 (5.81) | 12.92 (5.83) | < .001 | d = 0.20 |
| Baseline GAD-7 ≥ 10 | 5,396 (63.6) | 392 (68.7) | .015 | φ = .026 |
| Baseline PHQ-9 ≥ 10 | 5,326 (62.8) | 394 (69.0) | .003 | φ = .031 |
| Ever taken psychotropic medication | 5,625 (70.5) | 405 (73.8) | .101 | φ = .018 |
| Psychotropic medication in past 3 months | 4,610 (56.1) | 327 (58.9) | .200 | φ = .014 |
| Currently receiving other mental health treatment | 3,892 (45.9) | 259 (45.4) | .803 | φ = −.003 |
| Lifetime diagnosis: Yes | 3,011 (39.2) | 224 (43.2) | .070 | φ = .020 |

**Note.** Early dropout was defined as completing 0–1 lessons; engaged participants completed two or more lessons. Ns vary across variables due to routine-care missingness. Percentages are calculated within each engagement group using available cases for each variable. *p* values are based on independent-samples *t* tests for continuous variables and Pearson χ² tests for categorical variables. Effect sizes are Cohen’s *d* for continuous variables, Cramer’s *V* for categorical variables with more than two levels, and φ for binary categorical variables. Cohen’s *d* was calculated as early dropout minus engaged.

**Table S2**.
Screening characteristics by development and temporal validation period

| **Characteristic** | **TRAIN 2013–2021** | **TEST 2022–2024** | **Effect size** |
| --- | --- | --- | --- |
| Age, M (SD) | 37.51 (13.11) | 39.93 (14.04) | d = 0.18 |
| PHQ-9 baseline, M (SD) | 11.92 (5.76) | 11.71 (5.97) | d = -0.04 |
| GAD-7 baseline, M (SD) | 11.76 (5.15) | 11.50 (5.25) | d = -0.05 |
| Gender, % | Women 75.1; men 24.1; other 0.8 | Women 70.1; men 27.6; other 2.2 | V = .073 |
| Education, % | High school or less 20.5; post-high school 44.5; university 34.9 | High school or less 21.1; post-high school 44.2; university 34.7 | V = .007 |
| Ethnicity, % | White 88.8; Indigenous 5.2; other 6.0 | White 81.9; Indigenous 7.2; other 10.9 | V = .096 |
| Geographic location, % | Rural 31.1; small/medium city 19.9; large city 49.0 | Rural 35.2; small/medium city 9.5; large city 55.3 | V = .125 |
| Relationship status, % | Single 27.5; married/common-law 62.2; separated/divorced/widowed 10.3 | Single 29.0; married/common-law 60.1; separated/divorced/widowed 10.8 | V = .019 |
| Employment status, % | Paid work 59.9; household/homemaker 12.0; retired 5.4; student 6.4; unfit for work 8.2; no paid work/other 8.1 | Paid work 59.2; household/homemaker 12.1; retired 7.7; student 4.8; unfit for work 9.9; no paid work/other 6.3 | V = .065 |
| Current mental health treatment, % yes | 45.3 | 47.3 | φ = .018 |
| PHQ-9 ≥ 10 at baseline, % | 64.0 | 61.1 | φ = -.027 |
| GAD-7 ≥ 10 at baseline, % | 64.4 | 62.7 | φ = -.016 |

Note. TRAIN = development period; TEST = temporal validation period. Values are based on the primary model complete-case sample (N = 9,042; TRAIN n = 6,495; TEST n = 2,547). Continuous variables are summarized as means and standard deviations. Categorical variables are summarized as percentages within period. Effect sizes are Cohen’s d for continuous variables, Cramer’s V for multi-category variables, and phi (φ) for binary variables. Positive d values indicate higher values in TEST than TRAIN.

**Table S3**.
Completion calibration by decile in the temporal test set

| **Test-set decile** | **n** | **Mean predicted P(completion)** | **Observed**  **completion rate** | **Observed − Predicted** |
| --- | --- | --- | --- | --- |
| 1, lowest | 254 | 0.483 | 0.382 | -0.101 |
| 2 | 254 | 0.553 | 0.535 | -0.018 |
| 3 | 256 | 0.593 | 0.547 | -0.046 |
| 4 | 256 | 0.626 | 0.578 | -0.048 |
| 5 | 252 | 0.657 | 0.599 | -0.058 |
| 6 | 254 | 0.684 | 0.665 | -0.019 |
| 7 | 256 | 0.712 | 0.641 | -0.071 |
| 8 | 255 | 0.745 | 0.690 | -0.055 |
| 9 | 255 | 0.787 | 0.765 | -0.022 |
| 10, highest | 255 | 0.850 | 0.835 | -0.014 |
| Total | 2,547 | 0.669 | 0.624 | -0.045 |

Note. Deciles were created in the temporal test sample using TRAIN-derived predicted probabilities for completion. Negative values in the Observed − Predicted column indicate overprediction.

**Table S4**.
Early-dropout calibration by quintile in the temporal test set

| **Test-set quintile** | **n** | **Mean predicted**  **P(early dropout)** | **Observed early dropout rate** | **Observed − Predicted** |
| --- | --- | --- | --- | --- |
| 1, lowest | 510 | 0.025 | 0.045 | 0.020 |
| 2 | 509 | 0.039 | 0.053 | 0.014 |
| 3 | 511 | 0.052 | 0.055 | 0.003 |
| 4 | 508 | 0.068 | 0.077 | 0.009 |
| 5, highest | 509 | 0.110 | 0.116 | 0.006 |
| Total | 2,547 | 0.059 | 0.069 | 0.010 |

Note. Quintiles were created in the temporal test sample using TRAIN-derived predicted probabilities for early dropout. Positive values in the Observed − Predicted column indicate underprediction.

**Table S5**.
Secondary multinomial logistic regression predicting three-category engagement stage

**Panel A**. Early dropout (0–1 lessons) versus partial engagement (2–4 lessons)

| Predictor (reference) | B | SE | OR | 95% CI | *p* |
| --- | --- | --- | --- | --- | --- |
| Age, per year | -0.009 | 0.005 | 0.991 | [0.981, 1.001] | .094 |
| Gender: Men vs Women | 0.034 | 0.110 | 1.034 | [0.834, 1.283] | .758 |
| Gender: Other vs Women | -0.471 | 0.445 | 0.625 | [0.261, 1.495] | .290 |
| Education: Post-high school vs High school or less | -0.046 | 0.117 | 0.955 | [0.759, 1.202] | .698 |
| Education: University degree vs High school or less | -0.216 | 0.135 | 0.805 | [0.619, 1.049] | .108 |
| Ethnicity: Indigenous vs White | 0.275 | 0.163 | 1.316 | [0.957, 1.810] | .091 |
| Ethnicity: Other vs White | 0.289 | 0.157 | 1.336 | [0.982, 1.817] | .065 |
| Relationship: Married/common law vs Single/never married/dating | -0.115 | 0.113 | 0.891 | [0.715, 1.111] | .307 |
| Relationship: Separated/divorced/widowed vs Single/never married/dating | -0.260 | 0.193 | 0.771 | [0.528, 1.124] | .176 |
| Employment: Homemaker/household vs Paid work | -0.026 | 0.152 | 0.974 | [0.723, 1.312] | .862 |
| Employment: Retired vs Paid work | 0.575 | 0.303 | 1.777 | [0.981, 3.219] | .058 |
| Employment: Student vs Paid work | -0.011 | 0.182 | 0.989 | [0.692, 1.413] | .950 |
| Employment: Unfit for work due to health vs Paid work | -0.036 | 0.183 | 0.965 | [0.674, 1.381] | .846 |
| Employment: No paid work for other reasons vs Paid work | 0.106 | 0.165 | 1.112 | [0.804, 1.538] | .520 |
| Location: Small-to-medium city vs Small rural | 0.054 | 0.139 | 1.055 | [0.803, 1.386] | .701 |
| Location: Large city vs Small rural | 0.056 | 0.109 | 1.058 | [0.855, 1.310] | .604 |
| Current mental health treatment: Yes vs No | 0.002 | 0.095 | 1.002 | [0.832, 1.207] | .979 |
| PHQ-9 clinical baseline: Yes vs No | -0.082 | 0.112 | 0.921 | [0.740, 1.148] | .464 |
| GAD-7 clinical baseline: Yes vs No | 0.125 | 0.111 | 1.133 | [0.912, 1.407] | .259 |

**Panel B**. Completion (5 lessons) versus partial engagement (2–4 lessons)

| Predictor (reference) | B | SE | OR | 95% CI | p |
| --- | --- | --- | --- | --- | --- |
| Age, per year | 0.026 | 0.003 | 1.027 | [1.022, 1.032] | < .001 |
| Gender: Men vs Women | -0.049 | 0.057 | 0.953 | [0.851, 1.066] | .398 |
| Gender: Other vs Women | -0.069 | 0.212 | 0.934 | [0.616, 1.416] | .746 |
| Education: Post-high school vs High school or less | 0.035 | 0.064 | 1.036 | [0.914, 1.174] | .582 |
| Education: University degree vs High school or less | 0.243 | 0.069 | 1.275 | [1.113, 1.460] | < .001 |
| Ethnicity: Indigenous vs White | -0.422 | 0.099 | 0.656 | [0.540, 0.797] | < .001 |
| Ethnicity: Other vs White | -0.219 | 0.091 | 0.803 | [0.672, 0.960] | .016 |
| Relationship: Married/common law vs Single/never married/dating | 0.057 | 0.060 | 1.059 | [0.940, 1.192] | .345 |
| Relationship: Separated/divorced/widowed vs Single/never married/dating | -0.289 | 0.095 | 0.749 | [0.621, 0.903] | .002 |
| Employment: Homemaker/household vs Paid work | -0.047 | 0.077 | 0.954 | [0.821, 1.110] | .545 |
| Employment: Retired vs Paid work | 0.368 | 0.149 | 1.445 | [1.080, 1.933] | .013 |
| Employment: Student vs Paid work | 0.046 | 0.104 | 1.047 | [0.853, 1.284] | .663 |
| Employment: Unfit for work due to health vs Paid work | 0.092 | 0.091 | 1.097 | [0.918, 1.310] | .309 |
| Employment: No paid work for other reasons vs Paid work | 0.000 | 0.092 | 1.000 | [0.835, 1.198] | .998 |
| Location: Small-to-medium city vs Small rural | 0.030 | 0.072 | 1.030 | [0.894, 1.187] | .679 |
| Location: Large city vs Small rural | -0.008 | 0.056 | 0.992 | [0.890, 1.107] | .889 |
| Current mental health treatment: Yes vs No | 0.009 | 0.049 | 1.009 | [0.916, 1.111] | .862 |
| PHQ-9 clinical baseline: Yes vs No | -0.352 | 0.057 | 0.703 | [0.629, 0.786] | < .001 |
| GAD-7 clinical baseline: Yes vs No | 0.099 | 0.056 | 1.104 | [0.990, 1.233] | .076 |

**Note.** Secondary multinomial logistic regression estimated in the pooled primary complete-case sample (*N* = 9,042): early dropout, *n* = 571; partial engagement, *n* = 2,613; completion, *n* = 5,858. Partial engagement (2–4 lessons) was the reference outcome category. All predictors were entered simultaneously. Values are regression coefficients (*B*), standard errors (*SE*), odds ratios (OR = Exp[*B*]), and 95% confidence intervals (CI). Predictor reference categories were women for gender, high school education or less for education, White for ethnicity, single/never married/dating for relationship status, paid work for employment status, small rural location for geographic location, no current mental health treatment, and non-clinical baseline status on the PHQ-9 and GAD-7. The overall multinomial model was significant, χ²(38) = 469.12, *p* < .001, Nagelkerke *R*² = .063.

**Table S6**.

Adjusted associations with completion and early dropout within the weekly-assessment era, 2020–2021 versus 2022–2024

**Panel A**. Treatment completion

| **Predictor (reference)** | **2020–2021 OR** | **95% CI** | **p** | **2022–2024 OR** | **95% CI** | ***p*** |
| --- | --- | --- | --- | --- | --- | --- |
| Age, per year | 1.034 | [1.025, 1.043] | < .001 | 1.03 | [1.021, 1.039] | < .001 |
| Gender: Men vs Women | 1.013 | [0.826, 1.243] | 0.9 | 0.948 | [0.782, 1.150] | 0.59 |
| Gender: Other vs Women | 1.211 | [0.564, 2.601] | 0.624 | 0.992 | [0.571, 1.724] | 0.977 |
| Education: Post-high school vs High school or less | 0.961 | [0.770, 1.199] | 0.724 | 0.989 | [0.793, 1.234] | 0.925 |
| Education: University degree vs High school or less | 1.151 | [0.908, 1.458] | 0.246 | 1.38 | [1.084, 1.757] | 0.009 |
| Ethnicity: Indigenous vs White | 0.741 | [0.529, 1.037] | 0.081 | 0.622 | [0.454, 0.851] | 0.003 |
| Ethnicity: Other vs White | 1.024 | [0.743, 1.413] | 0.883 | 0.903 | [0.690, 1.182] | 0.459 |
| Relationship: Married/common law vs Single/never married/dating | 0.969 | [0.788, 1.191] | 0.765 | 0.998 | [0.811, 1.229] | 0.988 |
| Relationship: Separated/divorced/widowed vs Single/never married/dating | 0.665 | [0.473, 0.934] | 0.018 | 0.798 | [0.575, 1.108] | 0.178 |
| Employment: Homemaker/household vs Paid work | 1.008 | [0.798, 1.273] | 0.947 | 1.104 | [0.845, 1.442] | 0.47 |
| Employment: Retired vs Paid work | 1.007 | [0.629, 1.613] | 0.976 | 1.539 | [0.970, 2.442] | 0.067 |
| Employment: Student vs Paid work | 1.042 | [0.717, 1.513] | 0.83 | 1.027 | [0.690, 1.526] | 0.897 |
| Employment: Unfit for work due to health vs Paid work | 1.088 | [0.808, 1.467] | 0.578 | 1.546 | [1.142, 2.093] | 0.005 |
| Employment: No paid work for other reasons vs Paid work | 1.198 | [0.892, 1.611] | 0.231 | 1.003 | [0.711, 1.415] | 0.987 |
| Location: Small-to-medium city vs Small rural | 0.971 | [0.721, 1.306] | 0.844 | 0.904 | [0.666, 1.225] | 0.513 |
| Location: Large city vs Small rural | 1.064 | [0.887, 1.277] | 0.501 | 0.91 | [0.755, 1.097] | 0.323 |
| Current mental health treatment: Yes vs No | 0.958 | [0.810, 1.133] | 0.614 | 1.129 | [0.952, 1.340] | 0.162 |
| PHQ-9 clinical baseline: Yes vs No | 0.851 | [0.704, 1.028] | 0.094 | 0.6 | [0.492, 0.731] | < .001 |
| GAD-7 clinical baseline: Yes vs No | 0.998 | [0.828, 1.203] | 0.982 | 1.18 | [0.968, 1.437] | 0.101 |

**Panel B**. Early dropout

| **Predictor (reference)** | **2020–2021 OR** | **95% CI** | **p** | **2022–2024 OR** | **95% CI** | **p** |
| --- | --- | --- | --- | --- | --- | --- |
| Age, per year | 0.967 | [0.949, 0.984] | < .001 | 0.967 | [0.950, 0.984] | < .001 |
| Gender: Men vs Women | 1.060 | [0.726, 1.548] | 0.761 | 0.808 | [0.554, 1.178] | 0.267 |
| Gender: Other vs Women | 0.344 | [0.046, 2.592] | 0.3 | 0.928 | [0.358, 2.406] | 0.877 |
| Education: Post-high school vs High school or less | 1.023 | [0.696, 1.504] | 0.909 | 1.008 | [0.677, 1.498] | 0.97 |
| Education: University degree vs High school or less | 0.697 | [0.445, 1.091] | 0.115 | 0.723 | [0.460, 1.138] | 0.161 |
| Ethnicity: Indigenous vs White | 2.431 | [1.510, 3.912] | < .001 | 1.302 | [0.757, 2.239] | 0.34 |
| Ethnicity: Other vs White | 1.435 | [0.840, 2.454] | 0.186 | 1.772 | [1.136, 2.765] | 0.012 |
| Relationship: Married/common law vs Single/never married/dating | 0.778 | [0.543, 1.114] | 0.17 | 1.033 | [0.705, 1.513] | 0.868 |
| Relationship: Separated/divorced/widowed vs Single/never married/dating | 0.893 | [0.457, 1.744] | 0.739 | 1.162 | [0.617, 2.185] | 0.642 |
| Employment: Homemaker/household vs Paid work | 0.792 | [0.497, 1.263] | 0.328 | 0.86 | [0.523, 1.413] | 0.552 |
| Employment: Retired vs Paid work | 0.766 | [0.212, 2.773] | 0.685 | 1.872 | [0.812, 4.314] | 0.141 |
| Employment: Student vs Paid work | 0.796 | [0.438, 1.449] | 0.456 | 0.669 | [0.328, 1.363] | 0.268 |
| Employment: Unfit for work due to health vs Paid work | 0.756 | [0.419, 1.362] | 0.352 | 0.541 | [0.275, 1.065] | 0.075 |
| Employment: No paid work for other reasons vs Paid work | 0.822 | [0.487, 1.388] | 0.464 | 0.909 | [0.490, 1.686] | 0.762 |
| Location: Small-to-medium city vs Small rural | 1.78 | [1.079, 2.934] | 0.024 | 0.672 | [0.361, 1.253] | 0.211 |
| Location: Large city vs Small rural | 1.281 | [0.898, 1.828] | 0.172 | 0.934 | [0.664, 1.313] | 0.694 |
| Current mental health treatment: Yes vs No | 0.832 | [0.607, 1.140] | 0.252 | 0.908 | [0.660, 1.247] | 0.55 |
| PHQ-9 clinical baseline: Yes vs No | 1.239 | [0.861, 1.783] | 0.249 | 1.25 | [0.861, 1.815] | 0.24 |
| GAD-7 clinical baseline: Yes vs No | 1.209 | [0.843, 1.736] | 0.302 | 0.99 | [0.682, 1.438] | 0.96 |

**Note.** Analyses were restricted to the weekly-assessment era and compared participants screened in 2020–2021 (n = 2,507) with those screened in 2022–2024 (n = 2,547). Separate multivariable logistic regression models were fitted within each period using the same prespecified baseline predictors as the primary analyses. All predictors were entered simultaneously. In Panel A, the dependent variable was treatment completion (1 = completed all five lessons; 0 = completed fewer than five lessons). In Panel B, the dependent variable was early dropout (1 = completed 0–1 lesson; 0 = completed two or more lessons). OR = odds ratio; CI = confidence interval; PHQ-9 = Patient Health Questionnaire-9; GAD-7 = Generalized Anxiety Disorder-7. Odds ratios greater than 1 indicate higher odds of the respective outcome. Reference categories were women for gender, high school education or less for education, White for ethnicity, single/never married/dating for relationship status, paid work for employment status, small rural location for geographic location, no current mental health treatment, and non-clinical baseline status on the PHQ-9 and GAD-7. For completion, Nagelkerke R² was .063 in 2020–2021 and .096 in 2022–2024. For early dropout, Nagelkerke R² was .075 and .043, respectively.
