# Supplementary material for: Predicting completion and early dropout in routine-care internet-delivered cognitive behaviour therapy: A temporal validation study
Source: Internet Interv. 2026 Oct 12;46:101011. doi: 10.1016/j.invent.2026.101011 (PMC13682379; doi:10.1016/j.invent.2026.101011)
Supplement: Supplementary file 3 [file mmc3.docx]

**Supplementary Methods**

**Disclosure**: The equations and explanatory text in this Supplementary Methods section were drafted with assistance from ChatGPT (OpenAI) and reviewed for accuracy by the authors.

**Temporal validation design**

To assess whether engagement prediction models developed in earlier service years would remain accurate when applied to later service years, we used prespecified temporal validation. Models were estimated using screening data from 2013–2021 (TRAIN) and then applied unchanged to data from 2022–2024 (TEST).

**Fixed-coefficient scoring for logistic regression**

For each participant, the TRAIN logistic regression model yields a linear predictor (LP):

LP = β0 + β1X1 + β2X2 + … + βkXk

Predicted probability was then computed as:

p = 1 / (1 + e^(−LP))

In temporal validation, predicted probabilities for the TEST set were generated using the coefficients estimated in TRAIN (no refitting in TEST).

**Performance metrics**

Performance in the TEST set was summarized using accuracy, discrimination, and calibration.

*Accuracy (Brier score)*. The Brier score is the mean squared error of predicted probabilities:

Brier = (1/N) Σ (pi − yi)^2

Lower values indicate better overall accuracy. For context, a no-information model that predicts the observed event rate for all cases has Brier = p(1 − p), where p is the observed event rate in the evaluation sample.

*Discrimination (AUC)*. The area under the receiver operating characteristic curve (AUC) summarizes how well predicted probabilities separate outcome cases (y = 1) from non-cases (y = 0). AUC = 0.50 indicates chance-level separation; values closer to 1.00 indicate stronger separation.

*Calibration*. Calibration was examined by grouping predicted probabilities into quantiles (completion: deciles; early dropout: quintiles) and comparing the mean predicted probability with the observed outcome rate in each group (Supplementary Tables S3–S4).

Logistic recalibration. Calibration intercept and slope were estimated by fitting:

logit[Pr(Y = 1)] = α + γ × logit(p)

where logit(p) = ln[p/(1 − p)]. Here, p is the model-scored predicted probability for an individual; Pr(Y = 1) is the predicted probability of the event; Y is the observed binary outcome (1 = event, 0 = non-event); α is the calibration intercept (ideal ≈ 0); and γ is the calibration slope (ideal ≈ 1).

**Pooled scoring summary**

In addition to the temporal TEST results, we also reported pooled performance summaries from logistic regression models refitted in the full primary complete-case sample. These pooled summaries describe overall apparent performance across the full analytic period and do not constitute independent validation. Temporal TEST results provide the primary evidence of model transportability.

**Extreme-group decision-rule evaluation**

To illustrate potential utility of simple decision rules, we examined operating characteristics for prespecified extreme predicted-risk groups in the temporal TEST set. For completion, we evaluated the lowest decile of predicted completion probability (flagged as at risk for non-completion) and the highest decile (flagged as likely completers). For early dropout, we evaluated the highest-risk quintile.

For each rule, we computed:

• Prevalence = (TP + FN) / N

• Flag rate = (TP + FP) / N

• Sensitivity = TP / (TP + FN)

• Specificity = TN / (TN + FP)

• PPV = TP / (TP + FP)

• NPV = TN / (TN + FN)

where TP = true positives (flagged and the event occurred), FP = false positives (flagged and the event did not occur), FN = false negatives (not flagged and the event occurred), TN = true negatives (not flagged and the event did not occur), and N is the evaluation sample size. PPV is the positive predictive value and NPV is the negative predictive value. PPV depends strongly on outcome prevalence and is expected to be modest for rare outcomes such as early dropout.

**References**

Brier, G. W. (1950). Verification of forecasts expressed in terms of probability. *Monthly Weather Review*, 78(1), 1–3.

Van Calster, B., McLernon, D. J., van Smeden, M., Wynants, L., & Steyerberg, E. W. (2019). Calibration: The Achilles heel of predictive analytics. *BMC Medicine*, 17, 230.
